# Tabu search algorithm for the distance-constrained vehicle routing problem with split deliveries by order

**DOI:** 10.1371/journal.pone.0195457

**Published:** 2018-05-15

**Authors:** Yangkun Xia, Zhuo Fu, Lijun Pan, Fenghua Duan

**Affiliations:** 1 School of Traffic and Transportation Engineering, Central South University, Changsha, Hunan, China; 2 College of Management, Hunan Institute of Engineering, Xiangtan, Hunan, China; 3 Business School, Hunan University of Science and Technology, Xiangtan, Hunan, China; Beihang University, CHINA

## Abstract

The vehicle routing problem (VRP) has a wide range of applications in the field of logistics distribution. In order to reduce the cost of logistics distribution, the distance-constrained and capacitated VRP with split deliveries by order (DCVRPSDO) was studied. We show that the customer demand, which can’t be split in the classical VRP model, can only be discrete split deliveries by order. A model of double objective programming is constructed by taking the minimum number of vehicles used and minimum vehicle traveling cost as the first and the second objective, respectively. This approach contains a series of constraints, such as single depot, single vehicle type, distance-constrained and load capacity limit, split delivery by order, etc. DCVRPSDO is a new type of VRP. A new tabu search algorithm is designed to solve the problem and the examples testing show the efficiency of the proposed algorithm. This paper focuses on constructing a double objective mathematical programming model for DCVRPSDO and designing an adaptive tabu search algorithm (ATSA) with good performance to solving the problem. The performance of the ATSA is improved by adding some strategies into the search process, including: (a) a strategy of discrete split deliveries by order is used to split the customer demand; (b) a multi-neighborhood structure is designed to enhance the ability of global optimization; (c) two levels of evaluation objectives are set to select the current solution and the best solution; (d) a discriminating strategy of that the best solution must be feasible and the current solution can accept some infeasible solution, helps to balance the performance of the solution and the diversity of the neighborhood solution; (e) an adaptive penalty mechanism will help the candidate solution be closer to the neighborhood of feasible solution; (f) a strategy of tabu releasing is used to transfer the current solution into a new neighborhood of the better solution.

## Introduction

The vehicle routing problem (VRP) is an NP-Hard problem [1], which has received wide attention in the operations research and transportation fields. Since it was proposed by Dantzig in 1959 [1], the VRP has been widely applied in logistics distribution, railway scheduling, cruise control at sea, etc. [1–12]. In most existing VRP studies [3–40], the convention is that a customer demand can be completed by one vehicle at one time, which is an example of demand inseparable VRP. However, in order to improve the loading rate of vehicles and reduce the number of vehicles required in actual distribution, logistics enterprises sometimes allow the demand to be split and delivered by multiple vehicles [13]. Therefore, the vehicle routing problem with split loads (VRPSL) has practical significance.

Dror and Trudeau first [13] proposed one of the most basic types of the VRPSL in 1989, which is known as the VRP with split deliveries (VRPSD). They presented the definition of the VRPSD in their study and pointed out that reasonably splitting customer demand by multiple vehicles can not only reduce the number of vehicles required, but also the traveling cost. Later in 1994 [14], they proved that the VRPSD is an NP-hard problem and provided the sub-loop cancellation constraints. Their study enabled a group of researchers to enter the field of the VRP with split deliveries [15–32]. For example, Archetti et al. further simplified the difficulty of the VRPSD on this basis [15–19]. They established the classical integer programming model for the VRPSD (K-VRPSD model) by setting the vehicle capacity and customer demand to integer units, assuming that the demand of each customer can be arbitrarily split deliveries by unit and that the vehicle capacity is not less than the demand of each customer [15–19].

Following the Method of demand unitized splitting by Archetti et al. [15–16], Archetti et al. [17] used an enhanced branch and price-and-cut algorithm for solving the VRP with split deliveries and time windows (VRPSDTW). Moreno et al. [20] and Archetti C et al. [21–22] also studied the exact algorithms for solving the VRPSD. Yan et al. [23] proposed a daily VRP model for minimizing the total cost of replenishing inventory within a supply chain, and they used a classic two-step solution algorithm to solve the multi-trip VRP with split deliveries and soft time windows (VRPSDSTW). By testing of a real-world scale numerical example, they found their VRPSDSTW model is more effective than traditional VRP models. Nishi et al. [24] proposed a column generation based heuristic algorithm to solve a ship routing and scheduling problem for crude oil transportation with split deliveries. Based on the column generation method, they used an efficient heuristic algorithm to generate a feasible solution taking into account of practical constraints. Compared with the branch and bound algorithm and that of human operators, the column generation based heuristic algorithm has better performance [24].

As an NP-hard problem [25], many Scholars focused on metaheuristic algorithm for solving the VRPSD and its related extension types [25–27]. To solve the VRPSD, Aleman et al. [25] added the adaptive memory to their classical heuristic and metaheuristic algorithms. They carefully analyzed the constructive heuristic approach (CA), iterative constructive approach (ICA) and iterative constructive approach plus variable neighborhood descent (ICA+VND). Wilck et al. [26] developed two hybrid genetic algorithms to solve the VRPSD. Through numerical experiments, Wilck et al. [26] found that, with respect to the total travel distance and computer time, the genetic algorithm compares favorably versus a column generation method and a two-phase method [26]. Yin et al. [27] studied a practical ship scheduling problem for international crude oil transportation. They considered the problem as a VRPSD model, and they proposed a savings-based metaheuristic algorithm with lot sizing parameters and volume assignment heuristic to solve it. The computational results show that their heuristic algorithm is more effective than that of human operators [27].

Some extension types of the VRPSD were also solved by designing a series of new heuristic mechanisms into the metaheuristic algorithm. To solve the fleet size and mixed VRPSD with time windows (VRPSDTW), Belfiore et al. [28] proposed a scatter search (SS) approach for it. With a case study on the Great Sichuan Earthquake in China, Wang et al. [29] constructed a nonlinear integer open location-routing model for the relief distribution VRPSD with travel time, and they used the non-dominated sorting genetic algorithm and non-dominated sorting differential evolution algorithm to solve the problem. Rajappa et al. [30] designed an ant colony optimization (ACO) and hybrid metaheuristics algorithm to solve the VRPSD, while Han et al. [31] used a multi-start heuristic approach for solving the VRPSD with minimum delivery amounts (VRPSD-MDA). In order to provide cost savings to transportation and logistics operators, The VRP with simultaneous deliveries and pickups (VRPSDP) has attracted much research interest. Wang et al. [32] proposed a hybrid heuristic algorithm to solving the VRP of simultaneous deliveries and pickups with split loads and time windows (VRPSDPTW).

Though the method of demand unitized splitting is common in the existing VRPSD literature [13–32], some scholars still studied the VRP with discrete split deliveries (VRPDSD). Nakao et al. [33] proposed a new type of the VRPSD, that is, the VRPDSD. In their model of the VRPDSD, a customer’s demand consists of a set of items, each customer is allowed to be visited more than once, and each item is required to be serviced by exactly one vehicle. That is to say, each customer’s demand is allowed to split, but each item can not be split again. So its way of split deliveries is discrete. To solve the VRP with discrete split deliveries and time windows (VRPDSDTW), Salani et al. [34] designed a branch and price algorithm. Chen et al. [35] also considered the method of discrete split deliveries in their study of VRPSL, in which by using an a priori split strategy, that is, each customer demand is split into small pieces in advance, they constructed a novel approach to solve the new type of the VRPSL. Some representative literatures can be seen in Table 1.

**Table 1:** Some representative literatures on the VRPSL.

Type of VRPSL	Main method of splitting	Author	Type of algorithm	Main idea of algorithm
General VRPSD	Unitized splitting	Dror (1989) [13]	Classical heuristic algorithm	Two-stage main algorithm
General VRPSD	Unitized splitting	Dror (1994) [14]	Exact algorithm	Branch and bound algorithm
General VRPSD	Unitized splitting	Archetti (2006) [16]	Metaheuristic algorithm	Tabu search algorithm
General VRPSD	Unitized splitting	Aleman (2010) [25]	Classical heuristic and metaheuristic algorithm	Adaptive memory algorithm
General VRPSD	Unitized splitting	Archetti (2011) [21]	Exact algorithm	Column generation approach
General VRPSD	Unitized splitting	Wilck (2012) [26]	Metaheuristic algorithm	Hybrid genetic algorithm
General VRPSD	Unitized splitting	Archetti (2014) [22]	Exact algorithm	Branch-and-cut algorithm
General VRPSD	Unitized splitting	Berbotto (2014) [36]	Metaheuristic algorithm	Randomized granular tabu search heuristic
General VRPSD	Unitized splitting	Rajappa (2016) [30]	Metaheuristic algorithm	ant colony optimization (ACO) and hybrid metaheuristics algorithm
VRPSDTW	Unitized splitting	Ho (2004) [37]	Metaheuristic algorithm	Tabu search algorithm
VRPSDTW	Unitized splitting	Belfiore (2009) [38]	Metaheuristic algorithm	Scatter search algorithm
VRPSDTW	Unitized splitting	Archetti (2010) [17]	Exact algorithm	Enhanced branch and price-and-cut algorithm
VRPSDTW	Unitized splitting	Luo (2017) [39]	Exact algorithm	Branch and price-and-cut algorithm
VRPSDTW	Unitized splitting	Belfiore (2013) [28]	Metaheuristic algorithm	Scatter search aprrpoach
VRPSDSTW	Unitized splitting	Yan (2015) [23]	Classical heuristic algorithm	Classic two-step solution algorithm
VRPSDSTW	Unitized splitting	Chu (2017) [40]	Metaheuristic algorithm	Two-stage heuristic solution algorithm
VRPSD-MDA	Unitized splitting	Han (2016) [31]	Metaheuristic algorithm	Multi-start heuristic approach
VRPSDPTW	Unitized splitting	Wang (2013) [32]	Metaheuristic algorithm	hybrid heuristic algorithm
VRPDSD	Discrete split deliveries	Nakao (2007) [33]	Exact algorithm	Dynamic programming
VRPDSD	Discrete split deliveries	Chen (2017)[35]	Classical heuristic algorithm	Priori split strategy and column generation approach
VRPDSDTW	Discrete split deliveries	Salani (2011) [34]	Exact algorithm	Branch and price algorithm.

From the related model of VRPSD [13–35], the idea of demand continuously unitized splitting is often adopted in lots of the VRPSD literatures, while studies on the VRP with discrete split deliveries were rare [33–35]. However, in the practice of e-commerce logistics distribution, the way of split deliveries is usually discrete and goods are often assembled, packaged or delivered in "orders". Each customer’s demand usually contains multiple orders and a single order is unable to be split when the order is generated. Similarly, for utilities suppliers, supermarket distributors and courier deliveries, only the discrete splitting method, i.e. by order, is convenient, while unitized splitting is unsuitable. This is because the customers’ goods are often different in type, size, quality, etc. Therefore, this study on the VRP with split deliveries by order (VRPSDO) has theoretical and practical significance.

## Problem description and model

### Problem description

In the existing VRPSD models [13–40], vehicle capacity and customer time windows were addressed without taking “distance-constrained” variables into consideration. The main reason for this is that traditional long-distance freight transportation practice does not have restrictions on routing length and there is only an approximate term. This period is usually far beyond the actual need of the road transportation and the driver’s normal driving will certainly not impact delivery. Moreover, early short distance distribution enterprises were mainly based on extensive development mode. They generally considered minimizing the delivery cost and increasing the vehicle loading rate as much as possible. After all, considering the “distance-constrained” case may reduce vehicle loading rate. However, late VRP tries to preempt customer resources by adding time window restrictions so as to improve customer satisfaction in a buyer's market environment. However, practically speaking, there are many customers who do not have requirements for the time windows. When there is a maximum “distance-constrained” case, the time of vehicle arrival to customer points will not be too late and does not impact customer business. However, in a seller's market where distribution resources are in short supply, the initiative of setting the time window is in the hands of the distributor. Distribution companies only need to notify their customers in advance to pick up the goods within a specified period of time according to the arrival time at each point. In this way, the time windows given by the distributor does not need to be included in the constraint.

In logistics distribution practice, especially in urban environments, vehicles carry only a limited amount of fuel and cannot travel for longer when the fuel runs out. Moreover, continuous traveling over a certain mileage threshold will cause great wear and tear to the vehicle parts, such as tires, bearings, steel plates and engines, especially in high temperatures zones or seasons. Additionally, continuous long-distance traveling causes driver fatigue. Considering factors such as the driver's continuous working hours and the balanced workload of each distribution path, the traveling distance for each delivery vehicle cannot be too long and should be “distance-constrained”. Logistics enterprises can determine the maximum length of the road according to the actual situation. Based on the analysis above, the study of distance-constrained VRPSDO is of importance.

In this study, we consider a single depot or distribution center with a single vehicle type that is used for deliveries only. We studied the distance-constrained and capacitated VRPSDO (DCVRPSDO), which can be described as follows. The problem calls for the determination of a set of minimum-cost routes to be performed by a fleet of vehicles to serve a given set of customers with known demands, where each route originates and terminates at a single depot. The route length and load limit conditions are satisfied. The demand of each customer can be split and served by multiple vehicles. However, the splitting can only be made according to orders, which refers in this study to the minimum set of customer demand. In summary, DCVRPSDO has some assumptions, as shown in Table 2.

**Table 2:** Some assumptions of the DCVRPSDO.

Essential factor	Assumptions
Depot	A single depot with known location and sufficient vehicles.
Routing	The effect of road traffic is ignored. The depot to customer points and one customer point to another is directly reachable.
Customer	The location and demand of all customers are known. The demand of each customer can be split and delivered by multiple vehicles, with the traveling time between customers satisfying triangle inequality.
Vehicle	A single vehicle type whereby all vehicles have the same loading capacity and cannot be overloaded, with each vehicle having a route length constraint. The vehicles traveling speed is constant and they must also return to the original starting point after finishing their tasks.
Objective	Two levels of evaluation objectives were set. The first level is to minimize the number of vehicles used, while the second is to minimize the total traveling time of the used vehicles. The priority of the first level is higher than that of the second level.

The depot is numbered as 0, and customers are numbered as 1, 2, …, *N*. The notations are defined as in Table 3.

**Table 3:** Notations of the DCVRPSDO.

Parameters or variables	Notations
*K*	The number of vehicles used (the number of routes).
*L*	Maximum route length.
*Q*	Vehicle capacity.
*d*_*i*_	The demand of customer *i*.
*R*	The actual maximum number of orders of all customers.
*d*_*i*_^*r*^	The demand of the *r*^th^ order of customer *i*.
*x*_*ir*_^*k*^	If vehicle *k* delivers the *r*^th^ order of customer *i*, the value is 1; otherwise the value is 0.
*y*_*ij*_^*k*^	If vehicle *k* visits *j* immediately after visiting *i*, the value is 1; otherwise the value is 0.
*t*_*ij*_	The traveling distance from customer *i* directly to customer *j*.
*n*_*k*_	The customer order sequence of the *k*^th^ route.
*S*	A solution to the problem, *S* = (*η*_1_,*η*_2_, …,*η*_*K*_).
*S*^initial^	Initial solution.
*S*^neighbor^	Neighborhood solution generated by neighborhood exchange
*S*^candi^	Candidate solutions selected from neighborhood solutions.
*S*^now^	The current solution in each iteration.
*S*^best^	The best solution in each iteration.
*Best*{}	According to the two evaluation indices, a function used to select the better solution, details are stated later.
*A*	Candidate solution set composed of *S*^candi^.
^#^*S*^candi^.	Best candidate solution in set *A*.
*S*^non-tabu^	Non tabued solution in set *A*, *S*^non-tabu^∈*A*.
*B*	Non tabued solution set composed of *S*^non-tabu^, *S*^non-tabu^∈*B*, *B*⊆*A*.
^#^*S*^non-tabu^	Best non-tabu solution in set *B*, ^#^*S*^non-tabu^ = Best *S*^non-tabu^ |*S*^non-tabu^ϵ*B* }.
*S*^feasible^	Feasible candidate solution in set *A*, *S*^feasible^∈*A*.
*C*	Feasible candidate solution set composed of *S*^feasible^ in set *A*, *S*^feasible^∈*C*, *C*⊆*A*.
*P*	The number of neighborhood solutions.
*M*_*u*1_	The total iteration number.
*M*_*u*2_	The iteration number of the “best solution” remaining unchanged.
*M*_*u*3_	The total iteration time.
*N*_*u*1_	The upper limit of *M*_*u*1_.
*N*_*u*2_	The upper limit of *M*_*u*2_.
*N*_*u*3_	The upper limit of *M*_*u*3_.

### Mathematical model

The total cost corresponding to a feasible solution with fewer vehicles is usually lower. Therefore, solutions with fewer vehicles are usually better than those with more vehicles [41]. To facilitate the solution of this kind, two levels of evaluation objectives are set. The first level is to minimize the number of vehicles used, while the second is to minimize the total traveling time of vehicles. The priority of the first level is higher than that of the second level. The double objective mathematical model of the DCVRPSDO is as follows.

minK (1)

$$\min\;Z=\sum_{i=0}^{N}\sum_{j=0}^{N}\sum_{k=1}^{K}\left(t_{ij}\cdot y_{ij}^{k}\right)$$ (2)

$$\sum_{i=0}^{N}\sum_{j=0}^{N}\left(t_{ij}\cdot y_{ij}^{k}\right)\leq L,\;k=1,2,\ldots ,K$$ (3)

$$\sum_{i=1}^{N}\sum_{r=1}^{R}\left(d_{i}^{r}\cdot x_{ir}^{k}\right)\leq Q,\;k=1,2,‥‥,K$$ (4)

$$\sum_{k=1}^{K}x_{ir}^{k}\text{=}1,\;i=1,2,\ldots ,N;r=1,2,\ldots ,R$$ (5)

$$\sum_{k=1}^{K}\sum_{r=1}^{R}(d_{i}^{r}\cdot x_{ir}^{k})=d_{i},i=1,\ldots ,N$$ (6)

$$\sum_{i=0}^{N}\sum_{k=1}^{K}y_{ij}^{k}\geq 1,\;j=1,2,\ldots ,N$$ (7)

$$\sum_{i=0}^{N}y_{ie}^{k}\text{=}\sum_{j=0}^{N}y_{ej}^{k},\;\;e=1,2,‥,N;k=1,2,\ldots ,K$$ (8)

$$\sum_{k=1}^{K}\sum_{j=1}^{N}y_{0j}^{k}\text{=}K$$ (9)

$$\sum_{k=1}^{K}\sum_{i=1}^{N}y_{i0}^{k}\text{=}K$$ (10)

$$\sum_{i,j\in n\times n}y_{ij}^{k}\leq \text{|}n\text{|}-1,\;\;n=1,2,\ldots ,N;k=1,\ldots ,K$$ (11)

$$K\geq K_{\min}\text{=}\left\lfloor \sum_{i=1}^{N}d_{i}/Q\right\rfloor \text{+1}$$ (12)

$$y_{ij}^{k}\in \{0,1\},i,j=0,1,\ldots ,N;k=1,\ldots ,K$$ (13)

Eqs (1) and (2) are objective functions. Eqs (3) and (4) are the route length constraint and capacity constraint. Eq (5) guarantees that each order can only be delivered by one vehicle and that a single order cannot be further split. Eq (6) is the constraint that the customer demand can only by split by orders whereby the demand of each customer can be composed of several discrete orders. Eq (7) guarantees that each customer is visited at least once. Eqs (8)–(10) guarantee that the numbers of entering vehicles and exiting vehicles of intermediate vertexes are the same and all vehicles start from and return to the depot. Eq (11) can eliminate sub-loops and Eq (12) can estimate the lower bound of the number of vehicles required, where ⌊⌋ is rounded down. Eq (13) is 0 or 1 integer constraint.

## Design of adaptive tabu search algorithm

In the DCVRPSDO, allowing the demand to be split makes the number of combinations increase dramatically. In solving large-scale problems, the algorithm has more significant challenges. It is generally believed that the solution of the VRPSD is more difficult than the general VRP. Since VRPSD is also a NP-Hard problem [15], the solution of it is more difficult than general VRP. Exact algorithms such as the branch and bound method, cut plane, dynamic programming, column generation tangent plane, and the branch tangent plane methods [33–35], can only solve small and medium scaled problems. Therefore, metaheuristic algorithms, such as the tabu search algorithm (TSA), genetic algorithm (GA), ant colony algorithm (ACO), particle swarm optimization (PSO), bee colony algorithm (BCA) and so on are usually used to solve large-scale VRPs [40–53]. From the viewpoint of the publications [4–49], TSA is an efficient algorithm to solve VRPs.

TSA is an intelligent algorithm (metaheuristic algorithm) simulating human thinking process, and is essentially a kind of global optimization algorithm [6]. TSA has advantages of simplicity, adaptability, rapidity, accuracy and robustness [6]. The neighborhood searching ability of TSA is relatively strong. In this study, adaptive operation was added to the basic TSA, and the multi-neighborhood structure and tabu list re-initialization strategies were designed to improve its adaptive global optimization ability, thus forming an Adaptive TSA (ATSA).

### Solution representation and initial solution

A permutation of the depot 0 and custom orders (where order quantities are not zero) is used to represent a solution of the problem, where 0 can appear more than once, each time for a new route, while each custom order can only occur once. The vertexes between two 0 consist of a route. For example, in solution $S=(0d_{1}^{1}d_{1}^{2}d_{2}^{1}d_{3}^{1}d_{3}^{2}d_{3}^{3}0d_{2}^{2}d_{4}^{1}d_{4}^{2}d_{5}^{1}d_{6}^{1}0\cdot \cdot \cdot 0)$, the first two routes are $\left(0d_{1}^{1}d_{1}^{2}d_{2}^{1}d_{3}^{1}d_{3}^{2}d_{3}^{3}0\right)$ and $\left(0d_{2}^{2}d_{4}^{1}d_{4}^{2}d_{5}^{1}d_{6}^{1}0\right)$, respectively. The first route represents starting from depot 0, sequentially delivering the 1^st^ and 2^nd^ orders of customer 1, the 1^st^ order of customer 2, and the 1^st^, 2^nd^ and 3^rd^ orders of customer 3, and finally returning to depot 0.

It is generally believed that the better initial solution *S*^initial^ helps the TSA to find a good final solution, while a poor *S*^initial^ can easily cause slow convergence of the algorithm. Moreover, in solving specific problems, according to the problem characters, some heuristic algorithms can be adopted to generate *S*^initial^ with high quality in advance, and then use TSA for further search. Such staged processing methodologies can usually improve the final solution quality [41]. In this study, a simple but practical “nearest zero” (here zero refers to depot 0) heuristic mechanism is used to generate the feasible *S*^initial^. Particularly, all customers are sorted in ascending order according to their distance to depot 0. Then, under the constraints of vehicle capacity and maximum route length, the orders corresponding to customers are added into the vehicle routes according to such ascending order. When capacity *Q* and maximum route length *L* are violated, a new route starts. In constructing a feasible *S*^initial^, splitting is not considered, i.e., the orders of one customer are all put into the same route.

### Solution evaluation

Accepting infeasible solutions helps the algorithm to search for a better feasible solution in iterative steps [41]. In this study, an adaptive penalty mechanism is designed. We denote *δ*_*k*_ as the excess quantity of the *k*^th^ route length and *ε*_*k*_ as the overload of the *k*^th^ vehicle with values as shown in (14) and (15).

$$\delta_{k}=\max\{0,\;\sum_{i=0}^{N}\sum_{j=0}^{N}\left(t_{ij}\cdot y_{ij}^{k}\right)-L\},\;k=1,2,\ldots ,K$$ (14)

$$\varepsilon_{k}=\max[0,\;\sum_{i=1}^{N}\sum_{r=1}^{R}(d_{i}^{r}\cdot x_{ir}^{k})-Q],\;k=1,2,\ldots ,K$$ (15)

To enhance the ability of ATSA on jumping out of local optima, the algorithm is designed to accept the transform of infeasible solutions so that it can generate more feasible solutions by the transition of infeasible solutions. Therefore, the overall evaluation function *G* in DCVRPSDO is designed as (16).

$$G=P_{1}\cdot K+P_{2}\cdot F$$ (16)

$$F=Z+Z^{\prime }$$ (17)

In this study, the dual objectives are calculated by a hierarchical mode. Here, *P*_1_ and *P*_2_ are qualitative concepts representing priority *P*_1_>>*P*_2_. In other words, when using function Best{} to select ^#^*S*^non-tabu^ *S*^non-tabu^ ∈*B*) and *S*^feasible^ *S*^feasible^ ∈*C*), small *K* values are first considered and then small *F* values [41]. The ATSA sets the minimization of the number of vehicles used as the first objective because a decrease in the number of vehicles is consistent with a decrease in the traveling cost. Generally, the decrease in the number of vehicles is because the vehicle loading rate is increased, shortening traveling distance and avoiding detours. *Z*′ in evaluation function *F* represents the penalty cost of violating (3) and (4), as follows.

$$Z^{\prime }=\lambda \cdot [H_{1}\cdot (\sum_{k=1}^{K}\delta_{k})+H_{2}\cdot (\sum_{k=1}^{K}\varepsilon_{k})]$$ (18)

where

- *H*_1_ and *H*_2_ are the number of non-zero values of *δ*_*k*_ and *ε*_*k*_, respectively, representing the weighted penalty coefficients for route length overrun and vehicle overloading;
- λ is an adaptive penalty coefficient, with value range of λ∈[20, 2000] and initial value of 100. If five successive iterations have infeasible solutions, it is multiplied by two; while if five successive iterations have feasible solutions, it is divided by two. The setting of parameter λ is necessary for the adaptive optimization potential of the ATSA, more thoroughly searching in neighborhoods, guiding the algorithm to iterate between feasible solutions and infeasible solutions adaptively, enhancing the algorithm’s ability of jumping out of local optima and improving the robustness of the algorithm.

### Multi-neighborhood structure design

The number of neighborhood solutions *S*^neighbor^ generated from each iteration is assigned to *P* and the neighborhood solutions are selected to construct candidate solution set *A* (*K*≥*K*_min_). After set *A* is generated, *S*^now^ and *S*^best^ are selected for the next iteration from set *A*. It is stipulated that *S*^now^ is allowed to violate the constraints, while *S*^best^ must be a feasible solution. A neighborhood transform can be used to generate *S*^neighbor^. In a neighborhood transform, two different routes *R*_1_ and *R*_2_ are selected from *S*^now^ each time as neighborhood routes. In the ATSA, a perturbation rule with the maximum duration route (PRMDR) is designed. If the maximum route length in *S*^now^ exceeds *L*, then let it be the neighborhood route *R*_1_ and randomly select a route from others as *R*_2_. Otherwise, randomly select two routes as *R*_1_ and *R*_2_.

In the ATSA, a multi-neighborhood structure is designed, and a random neighborhood transform strategy is adopted to randomly select a neighborhood to transform *S*^now^. In the neighborhood transform, after selecting *R*_1_ and *R*_2_ according to the PRMDR method, a customer or order is randomly selected in the two routes for transformation. After each neighborhood operation, the front zero is kept if multiple zeros exist. The orders from the same customer in the same route are arranged orderly (i.e., combining orders in the same route), and checked for relevant constraints. The neighborhood transform randomly selects neighborhood operators from the following 6 types. For example, in solution $S=(0d_{1}^{1}d_{1}^{2}d_{2}^{1}\underline{d_{3}^{1}}d_{3}^{2}d_{3}^{3}0d_{2}^{2}d_{4}^{1}d_{4}^{2}\underline{d_{5}^{1}}d_{6}^{1}0\cdot \cdot \cdot 0)$, the underlines indicate the two randomly selected orders *j*_1_ ($d_{3}^{1}$) and *j*_2_ ($d_{5}^{1}$), and the results after the operation are as follows.

1) Vertexes exchange. Exchange the customers corresponding to orders *j*_1_ and *j*_2_, i.e. totally exchange the orders corresponding to the customers selected from the two routes, resulting in $S^{\prime }=(0d_{1}^{1}d_{1}^{2}d_{2}^{1}\underline{d_{5}^{1}}0d_{2}^{2}d_{4}^{1}d_{4}^{2}\underline{d_{3}^{1}}d_{3}^{2}d_{3}^{3}d_{6}^{1}0\cdot \cdot \cdot 0)$.
2) Insert vertex forward. Insert the customer corresponding to order *j*_1_ before the customer corresponding to order *j*_2_, resulting in $S^{\prime }=(0d_{1}^{1}d_{1}^{2}d_{2}^{1}0d_{2}^{2}d_{4}^{1}d_{4}^{2}\underline{d_{3}^{1}}d_{3}^{2}d_{3}^{3}\underline{d_{5}^{1}}d_{6}^{1}0\cdot \cdot \cdot 0)$.
3) Insert vertex backward. Insert the customer corresponding to order *j*_1_ after the customer corresponding to order *j*_2_, resulting in $S^{\prime }=(0d_{1}^{1}d_{1}^{2}d_{2}^{1}0d_{2}^{2}d_{4}^{1}d_{4}^{2}\underline{d_{5}^{1}}\underline{d_{3}^{1}}d_{3}^{2}d_{3}^{3}d_{6}^{1}0\cdot \cdot \cdot 0)$.
4) Vertex inversion. Invert the arrangement of the customers corresponding to orders *j*_1_ and *j*_2_, resulting in $S^{\prime }=(0d_{1}^{1}d_{1}^{2}d_{2}^{1}\underline{d_{5}^{1}}d_{4}^{2}d_{4}^{1}d_{2}^{2}0d_{3}^{3}d_{3}^{2}\underline{d_{3}^{1}}d_{6}^{1}0\cdot \cdot \cdot 0)$, and then combine the orders of the same route in *S'* resulting in $S^{\prime \prime }=(0d_{1}^{1}d_{1}^{2}d_{2}^{1}d_{2}^{2}\underline{d_{5}^{1}}d_{4}^{2}d_{4}^{1}0d_{3}^{3}d_{3}^{2}\underline{d_{3}^{1}}d_{6}^{1}0\cdot \cdot \cdot 0)$.
5) Order exchange. Exchange orders *j*_1_ and *j*_2_, resulting in $S^{\prime }=(0d_{1}^{1}d_{1}^{2}d_{2}^{1}\underline{d_{5}^{1}}d_{3}^{2}d_{3}^{3}0d_{2}^{2}d_{4}^{1}d_{4}^{2}\underline{d_{3}^{1}}d_{6}^{1}0\cdot \cdot \cdot 0)$.
6) Tails exchange. Exchange the customers corresponding to the route after orders *j*_1_ and *j*_2_, resulting in $S^{\prime }=(0d_{1}^{1}d_{1}^{2}d_{2}^{1}\underline{d_{5}^{1}}d_{6}^{1}0d_{2}^{2}d_{4}^{1}d_{4}^{2}\underline{d_{3}^{1}}d_{3}^{2}d_{3}^{3}0\cdot \cdot \cdot 0)$.

### Tabu rules and termination conditions

The design of tabu rules is the core of the TSA and the selection of tabu length has great effect on algorithm performance. To prevent the loop search in the early stage of the algorithm and improve the stochastic diversity in the late stage of the algorithm, a mixed tabu length is set based on several approaches in the literature [45]. In the first *N*_*u*0_ steps it is set to the fixed value of 16, and after *N*_*u*0_ steps, it is set to a random integer between 5 and 16, and *N*_*u*0_ = 500+15*N*.

A matrix tabu list is set with *N×N* elements, with tabu objects being customers. In each iteration, when a “vertex pair” (*i*, *j*) of a neighborhood operation (or its corresponding order) is selected, and its corresponding *S*^candi^ is about to be the *S*^now^ of the next iteration, the element (*i*, *j*) of the tabu list is filled with corresponding tabu length. The basic principle is that, after each iteration, the tabu length of the tabu object is subtracted by 1, until it is 0. Moreover, reasonable tabu operation can accelerate the search process.

To avoid excessive tabu, a “tabu releasing” strategy is set. If a “feasible candidate solution” *S*^feasible^ is better than current *S*^best^, such as “*K*(*S*^feasible^)< *K*(*S*^best^)” or “*K*(*S*^feasible^) = *K*(*S*^best^), *F*(*S*^feasible^)< *F*(*S*^best^)”, we then take this *S*^feasible^ as the new *S*^now^ and *S*^best^. Otherwise, we take ^#^*S*^non-tabu^ as the new *S*^now^. If all *S*^candi^ are tabued, i.e., *B* = Ø, then the tabu of the “best candidate solution” ^#^*S*^candi^ is released and let ^#^*S*^candi^ to be the new *S*^now^.

The tabu list has a short-term memory function and can avoid the repeated search when the tabu are appropriate. However, it may lose good solutions due to excessive tabu. In the iterative process, the routes of *S*^now^ are continuously updating. The following situation definite exists whereby different *S*^now^ select the same neighborhood exchange combination vertex pair (*i*, *j*) while the corresponding new solutions are different. Although these new solutions are not necessarily better than the current *S*^best^, these new solutions can transit to better *S*^neighbor^. Breaking the tabu and increasing the probability of these new solutions to be *S*^now^ is very desirable. To further prevent excessive tabu and improve the optimization ability of the ATSA, a tabu list re-initialization strategy is added. After *N*_*u*0_ steps, the tabu list is re-initialized every *m* iterations, i.e. the tabu list becomes an all-zero element matrix. In this study, we set *m* = 50.

Combining the methods in the literature [6–20], we set 3 stopping criteria for the ATSA. The algorithm ends if any of them is satisfied. The first is for the total iteration number (*M*_*u*1_) to reach the upper limit *N*_*u*1_, the second is for the iteration number of the “best solution” remaining unchanged (*M*_*u*2_) to reach the preset upper limit of iterations *N*_*u*2_ and the third is for the total iteration time (*M*_*u*3_) to reach the preset upper limit *N*_*u*3_.

### Algorithm description

According to the basic framework of the TSA, the ATSA algorithm can be described as follows.

Step 1: **Initialize**
Step 2: Read in the relevant data and parameter values.
Step 3: Generate feasible “initial solution” *S*^initial^ under distance and vehicle capacity constraints, according to “nearest zero” heuristic mechanism and take *S*^initial^ as the *S*^now^ and *S*^best^.
Step 4: ***While*** stopping criterion is not satisfied, ***do***
Step 5: ***While*** the number of *S*^neighbor^ is less than *P*, ***do***
Step 6: Randomly select one of the preset 6 neighborhood operators.
Step 7: Perform neighborhood transform operation according to selected rule for *S*^now^, and construct corresponding solution sets *A* and *C*.
Step 8: ***End***
Step 9: If a feasible candidate solution *S*^feasible^ (*S*^feasible^ ∈*A*) is better than current solution *S*^best^, take *S*^feasible^ as the new *S*^now^ and *S*^best^. Otherwise, construct solution set *B* and take ^#^*S*^non-tabu^ (^#^*S*^non-tabu^∈*B*) as the new *S*^now^. If *B* = Ø, remove the tabu situation of ^#^*S*^candi^ and take it as the new *S*^now^.
Step 10: Update tabu list
Step 11: ***End*.**

### Algorithm complexity

Denote the iteration steps of the algorithm as *I*, the number of neighborhood solutions generated by a single iteration as *P* and the problem solution as *wN* (since the number of orders is larger than that of customers). In the above described algorithm, the computation time mainly concentrates on iterations, the generation of neighborhood solutions and the selection of new *S*^now^ and new *S*^best^. In each iteration, the following several steps belong to sequential structure and the most complicated computation is in the stage of neighborhood solution generation of neighborhood search (including neighborhood exchange) with complexity *P·Ο(*(*wN*)^2^). The total algorithm complexity is *IP·Ο(*(*wN*)^2^). It is clear that the complexity of the distance-constrained ATSA is in proportion with the square of the problem scale *wN* and both the number of iterations and solutions impact computation time. This indicates that the complexity of our method is on the same order of magnitude of ordinary heuristic algorithms such as nearest neighbor insertion method, 2-exchange method, etc. [6] and is relatively reasonable. Moreover, it is clear from the algorithm complexity that *I* and *P* have impact on computation time. For a problem with a determined scale, when the requirement for result accuracy is not high, the computation time can be shortened by reducing the values of *I* and *P*.

## Results and discussion

Currently, there have not been benchmark examples of demand split by order with distance constraints. In this study, 7 large scale capacitated VRP (CVRP) numerical examples provided by Christofides et al. [2,25] were used to construct the testing examples (All relevant data are within the paper and the Supporting Information file S1 Appendix). They consisted of 50–199 customers. In the above examples, the distance constraints were added into and the customer demand quantities were randomly split into 1 to 4 orders, forming the numerical examples of the DCVRPSDO. The data of each example is listed in Table 4.

**Table 4:** The data of the test problems.

	a1	a2	a3	a4	a5	a6	a7
*N*	50	75	100	150	199	120	100
*Q*	160	140	200	200	200	200	200
*L*	180	144	160	200	220	220	220
*K*_min_	5	10	8	12	16	7	10

In the studies of the VRP with demand split by units (which belongs to classical K-VRPSD) by Archetti et al. [16] and Aleman and Hill et al. [2, 25], the above-mentioned 7 original examples without distance constraints were tested. The difference between their examples and those in this study lies in “distance constraints”. The results can be directly compared with the results of this study. The 7 numerical examples are numbered by a1, a2…a7 according to the order given by Aleman and Hill et al. [2, 25], where a part of demand data of a7 with 100 customers are listed in Table 5.

**Table 5:** Customer demand data for a7.

Vertex No.	Demand	Order No.			
		1	2	3	4
1	10	10	0	0	0
2	30	7	2	21	0
…	…				
37	20	3	17	0	0
…	…				
69	10	2	8	0	0
…	…				
100	20	13	7	0	0

### Results

In this study, Matlab2014a was used to implement the proposed ATSA and it was tested on a LENOVOV3000 with 2.40GHz AMD CPU and 4GB RAM. To make the algorithm suitable for problems of different scales without increasing the computational complexity too much, the relevant parameter values were set to linear to customer scale *N* with certain cardinality. The detailed parameter settings are as follows: *N*_*u*1_ = 4 000+100*N*, *N*_*u*2_ = 3 000+5*N*, *N*_*u*3_ = 400+12*N* and *P* = 50+*N*. According to the characters of the original examples, the Euclidean distance between vertexes represents the traveling time between vertexes. Each example was tested 8 times and the best results were used.

The results demonstrated that the convergence of the 7 examples was relatively good. The time for final solutions by the program was within 149.73–1773.49 seconds, which is acceptable. Apart from example a5 in which an additional vehicle was used, all examples reduced the number of vehicles used to the minimum.

### Comparative analysis

Archetti et al. designed a TSA in [12], but the test *Z* value of the numerical examples was not given. However, Aleman and Hill et al. [2, 30] used the same examples as those of Archetti et al. and gave the *Z* value results. Aleman and Hill et al. [2] carefully analyzed the constructive heuristic approach (CA), iterative constructive approach (ICA) and iterative constructive approach plus variable neighborhood descent (ICA+VND). CA constructed a route angle control (RAC) strategy, which was similar to the scanning algorithm with customers' demand split by angle, and used the cheapest customer insertion to optimize routings. This had relatively good solutions at first, but solutions degraded during the late insertions. ICA was based on CA and added an iterative searching function. It was used to optimize the degraded solutions of CA due to late customer insertions. ICA+VND algorithms adopted two stages to solve problems, whereby the results of ICA were used as the initial solutions of VND to obtain better solutions. It is clear from the analysis by Aleman and Hill et al. in [2] that CA belongs to heuristic algorithm, and ICA and ICA+VND belong to intelligent algorithms. The comparative studies combined the advantages of classical heuristic algorithms and the character is that the computation speed during the early stage is fast. But the algorithm robustness is unsatisfactory and is easy to be trapped local optima.

In many large-scale distribution VRP practices, enterprises are usually willing to sacrifice computation time to improve solution quality. In large-scale periodic distribution services, a percentage point increase in the solution quality would result in huge annual savings. To obtain high quality solutions, Aleman et al. [25] designed ICA+VND with Diversification (iVNDiv for short) based on ICA+VND. It mainly added a classification solution comparison mechanism of multi-starting solutions and an increased iteration number based on ICA+VND. In each iteration, the best solutions were kept from each class of solutions. In this study, the results of CA, ICA, ICA+VND and iVNDiv given by Aleman and Hill et al. [2, 25] are used for comparative analysis, with the comparison results of *Z* values for each example shown in Table 6 and the corresponding solution time shown in Table 7.

**Table 6:** Comparison results for distance of the ATS with others in the literature.

Pr.	ATSA	CA		ICA		ICA+VND		iVNDiv	
	*Z*	*Z*	IMP	*Z*	IMP	*Z*	IMP	*Z*	IMP
a1	**524.61**	578.83	10.34	568.67	8.40	540.82	3.09	**524.61**	0.00
a2	**843.55**	899.11	6.59	889.05	5.39	880.28	4.35	851.24	0.91
a3	**833.67**	873.46	4.77	863.18	3.54	854.13	2.45	852.74	2.29
a4	**1 062.94**	1 121.33	5.49	1 108.97	4.33	1 088.91	2.44	1 074.11	1.05
a5	**1 364.63**	1 412.18	3.48	1 412.18	3.48	1 390.55	1.90	1 368.67	0.30
a6	**1 144.41**	1 257.48	9.88	1 257.48	9.88	1 223.28	6.89	1 201.83	5.02
a7	**819.56**	827.59	0.98	826.03	0.79	824.82	0.64	824.78	0.64

Note: IMP represents the percentage of the comparative literature value *Z* higher than the ATS. The bold data represents the best value.

**Table 7:** Comparison results for CPU time of the ATS with others in the literature.

Pr.	ATSA	CA	ICA	ICA+VND	iVNDiv
	CPU	CPU	CPU	CPU	CPU
a1	149.73	0.08	2.69	10.89	54.91
a2	645.97	0.06	3.25	9.81	83.28
a3	1 197.84	0.16	9.34	43.50	319.33
a4	1 773.49	0.33	20.77	129.23	1 361.16
a5	1 369.17	0.55	26.66	534.83	3 284.64
a6	630.50	0.41	20.27	257.30	3 414.41
a7	645.10	0.11	6.20	21.02	126.08

Note: CPU running units are seconds.

Classical heuristic algorithms are adopted in the neighborhood searching process in the comparative studies and it is shown that their solution speed is fast during the early stage, but it is easy to get trapped in a local optimum during the late stage. After the iteration proceeds to a certain extent, the algorithm cannot jump out no matter how long it runs. For example, iVNDiv was improved based on ICA+VND with good solution quality during the early stage, but for large numerical examples (such as a5 and a6), the improvement after 3 000s was insignificant. The proposed ATSA uses the multi-neighborhood random searching rule in the iterative searching process with several robustness improvement strategies that enhanced the global optimization ability of the algorithm.

For the number of vehicles required, the proposed ATSA adopts a dynamic descending method. Apart from the first example (a5) which exceeded the minimum number of vehicles by 1, all examples reduced the results to the minimum number of vehicles used as the comparative algorithms did. This does not mean that the proposed ATSA is weaker than comparative algorithms in terms of reducing the number of vehicles. The comparative algorithms can arbitrarily split demand by units without distance constraints. Therefore, comparative algorithms can use a fixed minimum number of vehicles to search the shortest distance. However, the constraints in this study are more complicated. If the fixed minimum number of vehicles is used, there may be no feasible solution. Therefore, using the dynamic descending method for the number vehicles has better versatility compared with fixed ones, and the ability of reducing the number of vehicles for the proposed ATSA is also relatively strong.

In the aspect of traveling cost, apart from example (a1) for which iVNDiv and ATSA have the same best solutions, the *Z* values of all examples of the proposed ATSA are better than other algorithms, indicating that the ability of travel distance reduction is better than the comparative algorithms. The maximum saving ratios of ATSA to CA, ICA, ICA+VND, and iVNDiv for the examples are 10.34%, 9.88%, 6.89% and 5.02%, respectively, as shown in Table 6.

In the aspect of solution time, CA, ICA, and ICA+VND have relatively strong control over stopping criteria (solution time), with shorter solution time than the proposed algorithm. However, iVNDiv based on ICA+VND had a longer solution time than that of the proposed ATSA for the large numerical example and it also has a poorer solution quality. It is clear from Tables 6 and 7 that for heuristic algorithms of neighborhood searching with deterministic rules, although the solving speed during the early stage is fast, they are easily trapped in local optima during the late stage. Their global optimization ability is therefore weaker than that of ATSA with random neighborhood searching rules. Moreover, the object of the static VRP is to pursue the quality of the final solution. The final scheme does not have a high requirement for solution time. The planned distribution schemes do not need frequency changes like the dynamic VRP and it is workable if the solution time is within an acceptable range. Generally, the static VRP does not need to change the distribution scheme for a certain period, such as one month or one week.

It is clear that the algorithm complexity of ATSA is in proportion to the square of the problem scale, which is on the same order of magnitude of ordinary heuristic algorithms and relatively reasonable. For the same computing equipment, shortening the execution time of ATSA only requires reducing the numbers of iteration steps and candidate solutions (which may cause solution quality degradation). For different computing equipment, many factors may impact the specific solution time of the program, such as computer configuration and depreciation. Moreover, for the same computer configurations, desktops are much faster than notebooks. Aleman and Hill et al. [25] just described their platform as Pentium 4 2.8GHz CPU with 512MB RAM, without other descriptions of their platform. The equipment and stringency of the stopping criteria are different in the comparative studies and this study. Also, the algorithm complexity analysis was not given in the comparative studies and thus the computation time of each algorithm can only be compared roughly, as shown in Table 7. It is clear from Table 7 that the solution time of a4 (*N* = 150) is longer than that of a5 (*N* = 199) with a larger customer scale for proposed ATSA. For ICA+VND, the solution time of a6 (*N* = 120) is longer than that of a4 (*N* = 150) with larger customer scale. For iVNDiv, the solution time of a6 (*N* = 120) is longer than that of a4 (*N* = 150) and a5 (*N* = 199) with larger customer scale. The solution time of other algorithms is not in proportion to the customer scale. Therefore, it can be known that the solution time is not only related to the scale of customers in the problems, but also closely related to algorithm structures, geometric distribution of customers, demand distributions, constraints, etc.

The ATSA only allows the demand to be split by orders, which is stricter than the constraint of K-VRPSD of Aleman and Hill et al. [2, 25]. However, comparative algorithms without distance constraints are the relaxation of distance-constrained situations. From the viewpoint of solution space size, the solution sets of the test examples in this study are the subset of those in comparative algorithms. Theoretically, the comparative algorithms should obtain better results than that of the DCVRPSDO, bearing in mind that the finally results also depend on the optimization performance of the designed algorithm. It is clear from the test results that the overall solution quality of ATSA is higher than the 4 comparative algorithms, indicating the proposed ATSA has relatively strong optimization ability.

### Algorithm advantages and disadvantages

The distance-constrained ATSA with order-split belongs to a stochastic intelligent algorithm with advantages and disadvantages. Combined with the results of numerical tests, some advantages and disadvantages of the ATSA are described as Table 8.

**Table 8:** Some advantages and disadvantages of the ATSA.

Essential factor	Advantages	Disadvantages
Route length	Distance-constrained are added into the ATSA so that the length of the delivery route will be concentrated.	Adding distance restrictions easily reduces the vehicle loading rate and increases total cost.
Sensitivity of adaptive parameter	For maximum distance-constrained, an adaptive penalty mechanism is designed for the ATSA by accepting a part of the infeasible solutions for a transition to better feasible solutions and avoiding the premature convergence of the algorithm, which is conducive to improving the robustness of it. Moreover, experiments demonstrated that the algorithm was insensitive to the value of the adaptive coefficient λ∈[20, 2 200].	The adaptability of the algorithm is adjusted by λ, and the value of λ therefore has an impact on the solution quality. If a higher solution accuracy is expected, experiments are needed to determine the value range for λ.
demand-split strategy	The algorithms for the classical VRPs cannot directly solve the problem with demand split by order. In this study, the demand can only be split by order. We design a code format according to order split, where neighborhood operators perform unified operations for customers and orders, thereby reducing the difficulty of solving the order-split type problems.	Although the cost for demand split by order is theoretically lower than that of non-split, the difficulty of solving NP-Hard problems such as VRP increases significantly, and the requirement for the computational performance of algorithms is also higher.
Tabu strategy	The ATSA uses a matrix tabu list which can rapidly determine the tabu situation of customers. The ATSA uses a tabu list for short-term memory function, while uses a tabu releasing strategy, that is, re-initialization to avoid excessive tabu. It balances the two aspects of tabu and releasing, which can improve the robustness of the algorithm and improve the final solution quality.	when strategy of re-initialization is practiced, the solution time of numerical examples may be prolonged when the algorithm jumps out of a local optima.
Calculation time	The complexity of the ATSA is reasonable and on the same order of magnitude as ordinary heuristic algorithms. The multi-neighborhood structure is designed for the ATSA, which can simulate the distribution of extreme values in each neighborhood well and uses the random neighborhood operator selection rule. This is a progressive improvement method which can overcome the drawback of classical heuristic algorithms in which they can rapidly fall into the local optimum of neighborhoods and get stuck.	Since the improvement is achieved by randomly selecting neighborhoods, it depends on a certain probability. High quality solutions usually need more iteration steps and candidate solutions.

## Conclusions

The demand-split VRP belongs to the category of NP-Hard problems and has a wide value in the practice of distribution. Adding the distance-constrained aspect is helpful to improving the balance of routings. The splitting demand by orders in distribution is conducive to improving vehicle loading rate and reducing the total cost of delivery. In this study, The DCVRPSDO is studied and the corresponding double objective mathematical model is given. An ATSA is designed and the testing results demonstrate that ATSA can obtain relatively good final solutions in a reasonable calculation time. The DCVRPSDO is a new type of VRP. In this paper, a single order cannot be split again, maybe we can further study a mixed type of VRP, i.e. Some customers’ large order can be shipped by splitting but the small order remain stable.

In TSA, adopting a multi-neighborhood structure can simulate the extreme value distribution situations of the problem very well. Using the strategy of random neighborhood selection is helpful to realizing the access of multiple neighborhoods in several searching steps. The tabu vertexes can accelerate the optimization speed of the algorithm compared to the tabu solutions. Using mixed tabu length and adaptive penalty mechanism is conducive to improving the flexibility of neighborhood search and enhancing the richness of neighborhood solutions. Executing tabu list re-initialization strategy can avoid excessive tabu and improve the global optimization ability of the algorithm.

High quality optimal solutions usually need more iteration steps and candidate solutions. In other words, it can be inferred that in ATSA with random neighborhood selection rules, the values of *I* and *P* are larger than those of ATSA with traditional heuristic rules, which extends the calculation time of the search process. This also provides an idea of improvement, i.e. we can consider adding traditional heuristic rules into the local search of the ATSA. In addition, in the further study, we can consider adding more constraints into the DCVRPSDO, such as the multi-depots, multi-type vehicles, time windows, deliveries and pickups, dynamic vehicle routing, and so on.

## Supporting information

S1 Appendix Dataset. (PDF)

## References

[1] Dantzig G B, Ramser J H. The truck dispatching problem. Management Science. 1959; 6(1): 80–91.

[2] Aleman R E. A guided neighborhood search applied to the split delivery vehicle routing problem. Ohio: Wright State University, 2009.

[3] Jawarneh S, Abdullah S. Sequential insertion heuristic with adaptive bee colony optimisation algorithm for vehicle routing problem with time windows. Plos One. 2015; 10(7). https://doi.org/10.1371/journal.pone.0130224 10.1371/journal.pone.0130224 PMC4488911 26132158

[4] Hiermann G, Puchinger J, Ropke S, Ropke S, Hartl R F. The electric fleet size and mix vehicle routing problem with time windows and recharging stations. European Journal of Operational Research. 2016; 252(3): 995–1018.

[5] Fu Z, Wright M. Train plan model for British rail freight services through the channel tunnel. Journal of the Operational Research Society. 1994; 45(4): 384–391.

[6] Fu Z, Eglese R, Wright M. A branch-and-bound algorithm for finding all optimal solutions of the assignment problem. Asia-Pacific Journal of Operational Research. 2007; 24(6):831–839.

[7] Lai M, Tong X. A metaheuristic method for vehicle routing problem based on improved ant colony optimization and Tabu search. Journal of Industrial & Management Optimization. 2017; 8(2): 469–484.

[8] Wang Y, Ma X L, Lao Y T, Wang Y H. A fuzzy-based customer clustering approach with hierarchical structure for logistics network optimization. Expert Systems with Applications, 2014; 41(2): 521–534.

[9] Wang Y, Ma X L, Xu M Z, Liu Y, Wang YH. Two-echelon logistics distribution region partitioning problem based on a hybrid particle swarm optimization-genetic algorithm. Expert Systems with Applications, 2015, 42(12): 5019–5031.

[10] Luo Z, Qin H, Zhang D, Lim A. Adaptive large neighborhood search heuristics for the vehicle routing problem with stochastic demands and weight-related cost. Transportation Research Part E-Logistics and Transportation Review. 2016; 85: 69–89.

[11] Zhang D, Zou F, Li S, Zhou L. Green Supply Chain Network Design with Economies of Scale and Environmental Concerns. Journal of Advanced Transportation, 2017 https://doi.org/10.1155/2017/6350562

[12] Dong Z, Turnquist M. Combining service frequency and vehicle routing for managing supplier shipments. Transportation Research Part E: Logistics and Transportation Review. 2015; 79, 231–243. https://doi.org/10.1016/j.tre.2015.05.002

[13] Dror M, Trudeau P. Savings by split delivery routing. Transportation Science. 1989; 23(2): 141–145.

[14] Dror M, Laporte G, Trudeau P. Vehicle routing with split deliveries. Discrete Applied Mathematics. 1994; 50(3): 239–254.

[15] Archetti C, Mansini R, Speranza M G. Complexity and reducibility of the split delivery problem. Transportation Science. 2005; 39(2):182–187.

[16] Archetti C, Hertz A, Speranza M. A tabu search algorithm for the split delivery vehicle routing problem. Transportation Science. 2006; 40(1): 64–73.

[17] Archetti C, Bouchard M, Desaulniers G. Enhanced branch and price-and-cut for vehicle routing with split deliveries and time windows. Transportation Science. 2010; 45(3): 285–298.

[18] Archetti C, Feillet D, Gendreau M, Speranza M G. Complexity of the VRP and SDVRP. Transportation Research Part C: Emerging Technologies. 2011; 19(5):741–750.

[19] Archetti C, Speranza M G. Vehicle routing problems with split deliveries. International transactions in operational research. 2012; 19: 3–22. https://doi.org/10.1111/j.1475-3995.2011.00811.x

[20] Moreno L, De-aragao MP, Uchoa E. Improved lower bounds for the split delivery vehicle routing problem. Operations Research Letters. 2010; 38(4): 302–306.

[21] Archetti C, Bianchessi N, Speranza M. A column generation approach for the split delivery vehicle routing problem. Networks. 2011; 10: 241–254.

[22] Archetti C, Bianchessi N, Speranza MG. Branch-and-cut algorithms for the split delivery vehicle routing problem. European Journal of Operational Research. 2014; 238(3): 685–698.

[23] Yan S, Chu J C, Hsiao F Y, Huang H J. A planning model and solution algorithm for multi-trip split-delivery vehicle routing and scheduling problems with time windows. Computers & Industrial Engineering. 2015; 87: 383–393.

[24] Nishi T, Izuno T. Column generation heuristics for ship routing and scheduling problems in crude oil transportation with split deliveries. Computers & Chemical Engineering. 2014; 60(2): 329–338.

[25] Aleman R E, Zhang X H, Hill R R. An adaptive memory algorithm for the split delivery vehicle routing problem. Journal of Heuristics. 2010; 16(3): 441–473.

[26] Wilck J H IV, Cavalier T M. A genetic algorithm for the split delivery vehicle routing problem. American Journal of Operations Research. 2012; 2(2): 207–216.

[27] Yin S, Nishi T, Izuno T. A heuristic approach for international crude oil transportation scheduling problems. Journal of Advanced Mechanical Design, Systems, and Manufacturing, 2012; 6(5): 687–702.

[28] Belfiore P, Yoshizaki H T Y. Heuristic methods for the fleet size and mix vehicle routing problem with time windows and split deliveries. Computers & Industrial Engineering. 2013; 64(2): 589–601.

[29] Wang H, Du L, Ma S. Multi-objective open location-routing model with split delivery for optimized relief distribution in post-earthquake. Transportation Research Part E: Logistics and Transportation Review. 2014; 69(3): 160–179.

[30] Rajappa G P, Wilck J H IV, Bell J E. An ant colony optimization and hybrid metaheuristics algorithm to solve the split delivery vehicle routing problem. International Journal of Applied Industrial Engineering. 2016; 3(1):55–73.

[31] Han F W, Chu Y C. A multi-start heuristic approach for the split-delivery vehicle routing problem with minimum delivery amounts. Transportation Research Part E Logistics & Transportation Review. 2016; 88: 11–31.

[32] Wang Y, Ma X L, Lao Y T, Wang Y H, Mao H J. Vehicle routing problem: Simultaneous deliveries and pickups with split loads and time windows. Transportation Research Record: Journal of the Transportation Research Board. 2013; 2378: 120–128.

[33] Nakao Y, Nagamochi H. A DP-based heuristic algorithm for the discrete split delivery vehicle routing problem. Journal of Advanced Mechanical Design Systems & Manufacturing. 2007; 1(1): 217–226.

[34] Salani M, Vacca I. Branch and price for the vehicle routing problem with discrete split deliveries and time windows. European Journal of Operational Research. 2011; 213(3): 470–477.

[35] Chen P, Golden B, Wang X, Wasil E. A novel approach to solve the split delivery vehicle routing problem. International Transactions in Operational Research. 2017; 24: 27–41.

[36] Berbotto L, Garcia S, Nogales F. A randomized granular tabu search heuristic for the split delivery vehicle routing problem. Annals of Operations Research. 2014; 222: 153–173.

[37] Ho S C, Haugland D. A tabu search heuristic for the vehicle routing problem with time windows and split deliveries. Computers & Operations Research. 2004; 31(12): 1947–1964.

[38] Belfiore P C, Yoshizaki Y, Tsugunobu H. Scatter search for a real-life heterogeneous fleet vehicle routing problem with time windows and split deliveries in Brazil. European Journal of Operational Research. 2009; 199(3): 750–758.

[39] Luo Z X, Qin H, Zhu WB, Lim A. Branch and price and cut for the split-delivery vehicle routing problem with time windows and linear weight-related cost. Transportation Science. 2017; 51(2): 668–687.

[40] Chu J C, Yan S, Huang H J. A multi-trip split-delivery vehicle routing problem with time windows for inventory replenishment under stochastic travel times. Networks & Spatial Economics. 2017; 17(1): 41–68. https://doi.org/10.1007/s11067-015-9317-3

[41] Fu Z, Eglese R, Li YO. A new tabu search heuristic for the open vehicle routing problem. Journal of the Operational Research Society. 2005; 56(3), 267–274.

[42] Fu Z, Eglese R, Li L Y O. A unified tabu search algorithm for vehicle routing problems with soft time windows. Journal of the Operational Research Society. 2008; 59(5): 663–673.

[43] Mu Q, Fu Z, Lysgaard J, Eglese R. Disruption management of the vehicle routing problem with vehicle breakdown. Journal of the Operational Research Society. 2011; 62(4): 742–749.

[44] Mousavi M, Yap H J, Musa S N, Tahriri F, Dawal M Z S. Multi-objective AGV scheduling in an FMS using a hybrid of genetic algorithm and particle swarm optimization. Plos One. 2017; 12(3). https://doi.org/10.1371/journal.pone.0169817 10.1371/journal.pone.0169817 PMC5338791 28263994

[45] Xia Y K, Fu Z, & Xie J Y. Material distribution route planning for multiple automated guided vehicles with split deliveries by order. Computer Integrated Manufacturing Systems. 2017; 23(7), 1520–1528 https://doi.org/10.13196/j.cims.2017.07.017

[46] Fu Z, Liu W, & Qiu M. A tabu search algorithm for the vehicle routing problem with soft time windows and split deliveries by order. Chinese Journal of Management Science. 2017; 25(5), 78–86. https://doi.org/10.16381/j.cnki.issn1003-207x.2017.05.010

[47] Xia Y K, Fu Z. A tabu search algorithm for distribution network optimization with discrete split deliveries and soft time windows. Cluster Computing. 2018 3 29 https://doi.org/10.1007/s10586-018-2635-8

[48] Xia Y K, Fu Z. An adaptive tabu search algorithm for the open vehicle routing problem with split deliveries by order. Wireless Personal Communications. 2018 2 5 https://doi.org/10.1007/s11277-018-5464-4 10.1371/journal.pone.0195457 PMC5953470 29763419

[49] Xia Y K, Fu Z. Improved tabu search algorithm for open vehicle routing problem with soft time windows and satisfaction rate. Cluster Computing. 2018 2 17 https://doi.org/10.1007/s10586-018-1957-x

[50] Zhang D, Wang X, Li S, Ni N, Zhang Z. Joint optimization of green vehicle scheduling and routing problem with time-varying speeds. Plos One. 2018; 13(2). https://doi.org/10.1371/journal.pone.0192000 10.1371/journal.pone.0192000 PMC5821442 29466370

[51] Chan F, Shekhar P, Tiwari M. Dynamic scheduling of oil tankers with splitting of cargo at pickup and delivery locations: a Multi-objective Ant Colony-based approach. International Journal of Production. 2014; 52(24): 7436–7453.

[52] Yao B, Yu B, Hu P, Gao J, Zhang M. An improved particle swarm optimization for carton heterogeneous vehicle routing problem with a collection depot. Annals of Operations Research. 2016; 242(2): 1–18.

[53] Chen X, Kong Y, Dang L, Hou Y, Ye X. Exact and metaheuristic approaches for a bi-objective school bus scheduling problem. Plos One. 2016; 11(4). https://doi.org/10.1371/journal.pone.0132600 10.1371/journal.pone.0153614 PMC4824346 27054616

