# Supplementary material for: Tabu search algorithm for the distance-constrained vehicle routing problem with split deliveries by order
Source: PLoS One. 2018 May 15;13(5):e0195457. doi: 10.1371/journal.pone.0195457 (PMC5953470; doi:10.1371/journal.pone.0195457)
Supplement: S1 Appendix — (PDF) [file pone.0195457.s001.PDF]

S1 appendix. Dataset

a1

CAPACITY: 160

CUSTOMER QUANTITY: 50

MAXIMUM ROUTE LENGTH: 180

CUSTOMER

CUST NO.	XCOORD.	YCOORD.	DEMAND
0	30	40	0
1	37	52	7
2	49	49	30
3	52	64	16
4	20	26	9
5	40	30	21
6	21	47	15
7	17	63	19
8	31	62	23
9	52	33	11
10	51	21	5
11	42	41	19
12	31	32	29
13	5	25	23
14	12	42	21
15	36	16	10
16	52	41	15
17	27	23	3
18	17	33	41
19	13	13	9
20	57	58	28
21	62	42	8
22	42	57	8
23	16	57	16
24	8	52	10
25	7	38	28
26	27	68	7
27	30	48	15
28	43	67	14
29	58	48	6
30	58	27	19
31	37	69	11
32	38	46	12

33	46	10	23
34	61	33	26
35	62	63	17
36	63	69	6
37	32	22	9
38	45	35	15
39	59	15	14
40	5	6	7
41	10	17	27
42	21	10	13
43	5	64	11
44	30	15	16
45	39	10	10
46	32	39	5
47	25	32	25
48	25	55	17
49	48	28	18
50	56	37	10

----

a2

CAPACITY: 140

CUSTOMER QUANTITY: 75

MAXIMUM ROUTE LENGTH: 144

CUSTOMER

CUST NO.	XCOORD.	YCOORD.	DEMAND
0	40	40	0
1	22	22	18
2	36	26	26
3	21	45	11
4	45	35	30
5	55	20	21
6	33	34	19
7	50	50	15
8	55	45	16
9	26	59	29

10	40	66	26
11	55	65	37
12	35	51	16
13	62	35	12
14	62	57	31
15	62	24	8
16	21	36	19
17	33	44	20
18	9	56	13
19	62	48	15
20	66	14	22
21	44	13	28
22	26	13	12
23	11	28	6
24	7	43	27
25	17	64	14
26	41	46	18
27	55	34	17
28	35	16	29
29	52	26	13
30	43	26	22
31	31	76	25
32	22	53	28
33	26	29	27
34	50	40	19
35	55	50	10
36	54	10	12
37	60	15	14
38	47	66	24
39	30	60	16
40	30	50	33
41	12	17	15
42	15	14	11
43	16	19	18
44	21	48	17
45	50	30	21
46	51	42	27
47	50	15	19
48	48	21	20
49	12	38	5

50	15	56	22
51	29	39	12
52	54	38	19
53	55	57	22
54	67	41	16
55	10	70	7
56	6	25	26
57	65	27	14
58	40	60	21
59	70	64	24
60	64	4	13
61	36	6	15
62	30	20	18
63	20	30	11
64	15	5	28
65	50	70	9
66	57	72	37
67	45	42	30
68	38	33	10
69	50	4	8
70	66	8	11
71	59	5	3
72	35	60	1
73	27	24	6
74	40	20	10
75	40	37	20

----

a3

CAPACITY: 200

CUSTOMER QUANTITY: 100

MAXIMUM ROUTE LENGTH: 160

CUSTOMER

CUST NO.	XCOORD.	YCOORD.	DEMAND
0	35	35	0
1	41	49	10

2	35	17	7
3	55	45	13
4	55	20	19
5	15	30	26
6	25	30	3
7	20	50	5
8	10	43	9
9	55	60	16
10	30	60	16
11	20	65	12
12	50	35	19
13	30	25	23
14	15	10	20
15	30	5	8
16	10	20	19
17	5	30	2
18	20	40	12
19	15	60	17
20	45	65	9
21	45	20	11
22	45	10	18
23	55	5	29
24	65	35	3
25	65	20	6
26	45	30	17
27	35	40	16
28	41	37	16
29	64	42	9
30	40	60	21
31	31	52	27
32	35	69	23
33	53	52	11
34	65	55	14
35	63	65	8
36	2	60	5
37	20	20	8
38	5	5	16
39	60	12	31
40	40	25	9
41	42	7	5

42	24	12	5
43	23	3	7
44	11	14	18
45	6	38	16
46	2	48	1
47	8	56	27
48	13	52	36
49	6	68	30
50	47	47	13
51	49	58	10
52	27	43	9
53	37	31	14
54	57	29	18
55	63	23	2
56	53	12	6
57	32	12	7
58	36	26	18
59	21	24	28
60	17	34	3
61	12	24	13
62	24	58	19
63	27	69	10
64	15	77	9
65	62	77	20
66	49	73	25
67	67	5	25
68	56	39	36
69	37	47	6
70	37	56	5
71	57	68	15
72	47	16	25
73	44	17	9
74	46	13	8
75	49	11	18
76	49	42	13
77	53	43	14
78	61	52	3
79	57	48	23
80	56	37	6
81	55	54	26

82	15	47	16
83	14	37	11
84	11	31	7
85	16	22	41
86	4	18	35
87	28	18	26
88	26	52	9
89	26	35	15
90	31	67	3
91	15	19	1
92	22	22	2
93	18	24	22
94	26	27	27
95	25	24	20
96	22	27	11
97	25	21	12
98	19	21	10
99	20	26	9
100	18	18	17

----

a4

CAPACITY: 200

CUSTOMER QUANTITY: 150

MAXIMUM ROUTE LENGTH: 200

CUSTOMER

CUST NO.	XCOORD.	YCOORD.	DEMAND
0	35	35	0
1	41	49	10
2	35	17	7
3	55	45	13
4	55	20	19
5	15	30	26
6	25	30	3
7	20	50	5
8	10	43	9

9	55	60	16
10	30	60	16
11	20	65	12
12	50	35	19
13	30	25	23
14	15	10	20
15	30	5	8
16	10	20	19
17	5	30	2
18	20	40	12
19	15	60	17
20	45	65	9
21	45	20	11
22	45	10	18
23	55	5	29
24	65	35	3
25	65	20	6
26	45	30	17
27	35	40	16
28	41	37	16
29	64	42	9
30	40	60	21
31	31	52	27
32	35	69	23
33	53	52	11
34	65	55	14
35	63	65	8
36	2	60	5
37	20	20	8
38	5	5	16
39	60	12	31
40	40	25	9
41	42	7	5
42	24	12	5
43	23	3	7
44	11	14	18
45	6	38	16
46	2	48	1
47	8	56	27
48	13	52	36

49	6	68	30
50	47	47	13
51	49	58	10
52	27	43	9
53	37	31	14
54	57	29	18
55	63	23	2
56	53	12	6
57	32	12	7
58	36	26	18
59	21	24	28
60	17	34	3
61	12	24	13
62	24	58	19
63	27	69	10
64	15	77	9
65	62	77	20
66	49	73	25
67	67	5	25
68	56	39	36
69	37	47	6
70	37	56	5
71	57	68	15
72	47	16	25
73	44	17	9
74	46	13	8
75	49	11	18
76	49	42	13
77	53	43	14
78	61	52	3
79	57	48	23
80	56	37	6
81	55	54	26
82	15	47	16
83	14	37	11
84	11	31	7
85	16	22	41
86	4	18	35
87	28	18	26
88	26	52	9

89	26	35	15
90	31	67	3
91	15	19	1
92	22	22	2
93	18	24	22
94	26	27	27
95	25	24	20
96	22	27	11
97	25	21	12
98	19	21	10
99	20	26	9
100	18	18	17
101	37	52	7
102	49	49	30
103	52	64	16
104	20	26	9
105	40	30	21
106	21	47	15
107	17	63	19
108	31	62	23
109	52	33	11
110	51	21	5
111	42	41	19
112	31	32	29
113	5	25	23
114	12	42	21
115	36	16	10
116	52	41	15
117	27	23	3
118	17	33	41
119	13	13	9
120	57	58	28
121	62	42	8
122	42	57	8
123	16	57	16
124	8	52	10
125	7	38	28
126	27	68	7
127	30	48	15
128	43	67	14

129	58	48	6
130	58	27	19
131	37	69	11
132	38	46	12
133	46	10	23
134	61	33	26
135	62	63	17
136	63	69	6
137	32	22	9
138	45	35	15
139	59	15	14
140	5	6	7
141	10	17	27
142	21	10	13
143	5	64	11
144	30	15	16
145	39	10	10
146	32	39	5
147	25	32	25
148	25	55	17
149	48	28	18
150	56	37	10

----

a5

CAPACITY: 200
CUSTOMER QUANTITY: 199
MAXIMUM ROUTE LENGTH: 220
CUSTOMER

CUST NO.	XCOORD.	YCOORD.	DEMAND
0	35	35	0
1	41	49	10
2	35	17	7
3	55	45	13
4	55	20	19
5	15	30	26

6	25	30	3
7	20	50	5
8	10	43	9
9	55	60	16
10	30	60	16
11	20	65	12
12	50	35	19
13	30	25	23
14	15	10	20
15	30	5	8
16	10	20	19
17	5	30	2
18	20	40	12
19	15	60	17
20	45	65	9
21	45	20	11
22	45	10	18
23	55	5	29
24	65	35	3
25	65	20	6
26	45	30	17
27	35	40	16
28	41	37	16
29	64	42	9
30	40	60	21
31	31	52	27
32	35	69	23
33	53	52	11
34	65	55	14
35	63	65	8
36	2	60	5
37	20	20	8
38	5	5	16
39	60	12	31
40	40	25	9
41	42	7	5
42	24	12	5
43	23	3	7
44	11	14	18
45	6	38	16

46	2	48	1
47	8	56	27
48	13	52	36
49	6	68	30
50	47	47	13
51	49	58	10
52	27	43	9
53	37	31	14
54	57	29	18
55	63	23	2
56	53	12	6
57	32	12	7
58	36	26	18
59	21	24	28
60	17	34	3
61	12	24	13
62	24	58	19
63	27	69	10
64	15	77	9
65	62	77	20
66	49	73	25
67	67	5	25
68	56	39	36
69	37	47	6
70	37	56	5
71	57	68	15
72	47	16	25
73	44	17	9
74	46	13	8
75	49	11	18
76	49	42	13
77	53	43	14
78	61	52	3
79	57	48	23
80	56	37	6
81	55	54	26
82	15	47	16
83	14	37	11
84	11	31	7
85	16	22	41

86	4	18	35
87	28	18	26
88	26	52	9
89	26	35	15
90	31	67	3
91	15	19	1
92	22	22	2
93	18	24	22
94	26	27	27
95	25	24	20
96	22	27	11
97	25	21	12
98	19	21	10
99	20	26	9
100	18	18	17
101	37	52	7
102	49	49	30
103	52	64	16
104	20	26	9
105	40	30	21
106	21	47	15
107	17	63	19
108	31	62	23
109	52	33	11
110	51	21	5
111	42	41	19
112	31	32	29
113	5	25	23
114	12	42	21
115	36	16	10
116	52	41	15
117	27	23	3
118	17	33	41
119	13	13	9
120	57	58	28
121	62	42	8
122	42	57	8
123	16	57	16
124	8	52	10
125	7	38	28

126	27	68	7
127	30	48	15
128	43	67	14
129	58	48	6
130	58	27	19
131	37	69	11
132	38	46	12
133	46	10	23
134	61	33	26
135	62	63	17
136	63	69	6
137	32	22	9
138	45	35	15
139	59	15	14
140	5	6	7
141	10	17	27
142	21	10	13
143	5	64	11
144	30	15	16
145	39	10	10
146	32	39	5
147	25	32	25
148	25	55	17
149	48	28	18
150	56	37	10
151	22	22	18
152	36	26	26
153	21	45	11
154	45	35	30
155	55	20	21
156	33	34	19
157	50	50	15
158	55	45	16
159	26	59	29
160	40	66	26
161	55	65	37
162	35	51	16
163	62	35	12
164	62	57	31
165	62	24	8

166	21	36	19
167	33	44	20
168	9	56	13
169	62	48	15
170	66	14	22
171	44	13	28
172	26	13	12
173	11	28	6
174	7	43	27
175	17	64	14
176	41	46	18
177	55	34	17
178	35	16	29
179	52	26	13
180	43	26	22
181	31	76	25
182	22	53	28
183	26	29	27
184	50	40	19
185	55	50	10
186	54	10	12
187	60	15	14
188	47	66	24
189	30	60	16
190	30	50	33
191	12	17	15
192	15	14	11
193	16	19	18
194	21	48	17
195	50	30	21
196	51	42	27
197	50	15	19
198	48	21	20
199	12	38	5

----
a6
CAPACITY: 200
CUSTOMER QUANTITY: 120
MAXIMUM ROUTE LENGTH: 220
CUSTOMER

CUST NO.	XCOORD.	YCOORD.	DEMAND
0	10	45	0
1	25	1	25
2	25	3	7
3	31	5	13
4	32	5	6
5	31	7	14
6	32	9	5
7	34	9	11
8	46	9	19
9	35	7	5
10	34	6	15
11	35	5	15
12	47	6	17
13	40	5	13
14	39	3	12
15	36	3	18
16	73	6	13
17	73	8	18
18	24	36	12
19	76	6	17
20	76	10	4
21	76	13	7
22	78	3	12
23	78	9	13
24	79	3	8
25	79	5	16
26	79	11	15
27	82	3	6
28	82	7	5
29	90	15	9
30	84	3	11
31	84	5	10

32	84	9	3
33	85	1	7
34	87	5	2
35	85	8	4
36	87	7	4
37	86	41	18
38	86	44	14
39	86	46	12
40	85	55	17
41	89	43	20
42	89	46	14
43	89	52	16
44	92	42	10
45	92	52	9
46	94	42	11
47	94	44	7
48	94	48	13
49	96	42	5
50	99	46	4
51	99	50	21
52	83	80	13
53	83	83	11
54	85	81	12
55	85	85	14
56	85	89	10
57	87	80	8
58	87	86	16
59	90	77	19
60	90	88	5
61	93	82	17
62	93	84	7
63	93	89	16
64	94	86	14
65	95	80	17
66	99	89	13
67	37	83	17
68	50	80	13
69	35	85	14
70	35	87	16
71	44	86	7

72	46	89	13
73	46	83	9
74	46	87	11
75	46	89	35
76	48	83	5
77	50	85	28
78	50	88	7
79	54	86	3
80	54	90	10
81	10	35	7
82	10	40	12
83	18	30	11
84	17	35	10
85	16	38	8
86	14	40	11
87	15	42	21
88	11	42	4
89	18	40	15
90	21	39	16
91	20	40	4
92	18	41	16
93	20	44	7
94	22	44	10
95	16	45	9
96	20	45	11
97	25	45	17
98	30	55	12
99	20	50	11
100	22	51	7
101	18	49	9
102	16	48	11
103	20	55	12
104	18	53	7
105	14	50	8
106	15	51	6
107	16	54	5
108	28	33	12
109	33	38	13
110	30	50	7
111	13	40	7

112	15	36	8
113	18	31	11
114	25	37	13
115	30	46	11
116	25	52	10
117	16	33	7
118	25	35	4
119	5	40	20
120	5	50	13

----

a7

CAPACITY: 200

CUSTOMER QUANTITY: 100

MAXIMUM ROUTE LENGTH: 220

CUSTOMER

CUST NO.	XCOORD.	YCOORD.	DEMAND
0	40	50	0
1	45	68	10
2	45	70	30
3	42	66	10
4	42	68	10
5	42	65	10
6	40	69	20
7	40	66	20
8	38	68	20
9	38	70	10
10	35	66	10
11	35	69	10
12	25	85	20
13	22	75	30
14	22	85	10
15	20	80	40
16	20	85	40
17	18	75	20
18	15	75	20
19	15	80	10
20	30	50	10

21	30	52	20
22	28	52	20
23	28	55	10
24	25	50	10
25	25	52	40
26	25	55	10
27	23	52	10
28	23	55	20
29	20	50	10
30	20	55	10
31	10	35	20
32	10	40	30
33	8	40	40
34	8	45	20
35	5	35	10
36	5	45	10
37	2	40	20
38	0	40	30
39	0	45	20
40	35	30	10
41	35	32	10
42	33	32	20
43	33	35	10
44	32	30	10
45	30	30	10
46	30	32	30
47	30	35	10
48	28	30	10
49	28	35	10
50	26	32	10
51	25	30	10
52	25	35	10
53	44	5	20
54	42	10	40
55	42	15	10
56	40	5	30
57	40	15	40
58	38	5	30
59	38	15	10
60	35	5	20

61	50	30	10
62	50	35	20
63	50	40	50
64	48	30	10
65	48	40	10
66	47	35	10
67	47	40	10
68	45	30	10
69	45	35	10
70	95	30	30
71	95	35	20
72	53	30	10
73	92	30	10
74	53	35	50
75	45	65	20
76	90	35	10
77	88	30	10
78	88	35	20
79	87	30	10
80	85	25	10
81	85	35	30
82	75	55	20
83	72	55	10
84	70	58	20
85	68	60	30
86	66	55	10
87	65	55	20
88	65	60	30
89	63	58	10
90	60	55	10
91	60	60	10
92	67	85	20
93	65	85	40
94	65	82	10
95	62	80	30
96	60	80	10
97	60	85	30
98	58	75	20
99	55	80	10
100	55	85	20
